# Relationship between death anxiety, spiritual well-being, and social support in patients with gynecologic cancer

**DOI:** 10.1017/S1478951526102727

**Published:** 2026-06-03

**Authors:** Merve Sezer Daşçikaran, Evşen Nazik

**Affiliations:** 1 Department of Nursing Obstetrics and Gynecology Nursing, Institute of Health Sciences, Çukurova University, Adana, Turkey; 2 Department of Maternity and Gynecological Nursing, Çukurova University, Faculty of Health Sciences, Adana, Turkey

**Keywords:** Death anxiety, gynecologic cancer, gynecologic oncology, social support, spiritual well-being

## Abstract

**Objectives:**

This study was conducted to determine the relationship between death anxiety, spiritual well-being, and social support in patients with gynecological cancer.

**Methods:**

This descriptive study consisted of 519 patients with gynecological cancers. Data were collected using a “Personal Information Form,” the “Death Anxiety Scale,” the “Spiritual Well-Being Scale,” and the “Multidimensional Perceived Social Support Scale.”

**Results:**

The mean total score of the Death Anxiety Scale was 6.8 ± 2.95, the Spiritual Well-Being Scale was 31.16 ± 5.24, and the Multidimensional Perceived Social Support Scale was 62.46 ± 13.76. A positive correlation was found between the scores of the Multidimensional Perceived Social Support Scale and the Spiritual Well-Being Scale. However, death anxiety levels were not influenced by spiritual well-being or social support levels (*p* < 0.001). As the level of perceived social support increases, spiritual well-being also increases; however, no significant relationship was found between death anxiety and either spiritual well-being or perceived social support.

**Significance of results:**

The findings of this study highlight the importance of social support as a key factor associated with higher levels of spiritual well-being in women with gynecological cancer. Strengthening patients’ support systems may contribute to better psychological and spiritual adjustment during cancer treatment. Although death anxiety was not significantly related to either spiritual well-being or perceived social support, this result suggests that death anxiety may be influenced by other clinical or personal factors beyond these psychosocial variables. These findings provide guidance for developing supportive care programs that prioritize social support enhancement to improve overall patient well-being.

## Introduction

Cancer is the second leading cause of mortality on a global scale and is expected to rise to first place by 2030 (Türkiye Ministry of Health 2021). One in every 6 deaths on a global scale is caused by cancer, and this is 1 in every 5 deaths in Turkey (Akkoyun and Bekar 2024). Estimates by the International Agency for Research on Cancer show that 1 in every 5 women will develop cancer in the coming years, and 1 in every 10 will die from it (IARC 2024). It was reported that 95% of this increase would occur in developing countries (Wild et al. 2020). Based on the current data of GLOBOCAN, cervical cancer is the fourth, endometrial cancer is the sixth, and ovarian cancer is the eighth most common cancer among women (Globocan 2024). In Turkey, based on the data from the Cancer Department of the Ministry of Health (2018), endometrial cancer is the fourth, ovarian cancer is the sixth, and cervical cancer is the ninth among the top 10 cancers in women. Also, 10% of cancer-related deaths among women are caused by gynecological cancers (Ministry of Health 2018). Despite significant increases in survival rates among cancer patients due to advances in medicine, receiving a cancer diagnosis is a deeply traumatic experience for these patients. Cancer is a major stress factor that increases patients’ perception of the threat of death and, consequently, triggers various negative emotional responses (Zhao et al. 2018).

Women with gynecologic cancer frequently experience emotional challenges, including fear, uncertainty, anxiety, and concerns related to mortality (Akkanat Karagil and Harmancı 2022). The confrontation with a potentially life-limiting diagnosis may increase vulnerability to death anxiety. Death anxiety, defined as the emotional response to the awareness of mortality, is particularly relevant in life-threatening illnesses such as cancer. It is reported in the literature that cancer patients experience moderate death anxiety (Mansori et al. 2018; Feng et al. 2021; Ahmead et al. 2024; Wanglin 2024). Elevated death anxiety may be associated with impaired psychological adjustment and poorer health outcomes (Çal and Aydın Avcı 2023). However, theoretical approaches suggest that this anxiety can be alleviated by giving meaning and purpose to individuals’ lives. Studies have shown that an increased perceived level of meaning in life correlates with lower levels of death anxiety (Routledge and Juhl 2010; Lyke 2013).

In recent years, research has increasingly focused on spiritual well-being (SWB) as another variable influencing death anxiety in cancer patients. The World Health Organization (WHO) defined health as “*not only the absence of disease and disability but also a state of complete physical, social and spiritual well-being*” and drew attention to the importance of spiritual health (WHO 2024). Spirituality is the way a person gives meaning to his/her life and seeks answers within the environment, people, and nature he/she interacts with. Spirituality and religious beliefs can affect individuals’ perception of cancer and their health-related actions. Spirituality, which is effective in coping with cancer diagnosis and the treatment process, can affect the response to cancer and the approach to treatment positively. However, negative consequences such as the absence of divine power under the name of belief, not finding meaning in a cancer diagnosis, and thinking that one is being punished can also occur (Kurt 2021). Currently, religion and spirituality are gaining more and more importance in healthcare research. However, there is no universal definition or consensus for these 2 concepts. Religion generally refers to an “institutional structure,” and spirituality is more often considered an “individual orientation” (Akbolat 2022). Previous studies report that religion and spirituality are important factors affecting the quality of life in cancer patients (Akbolat 2022; Kurt 2021; Özdoğan 2021). Hajihasani and Naderi (2021) reported that spiritual health status reduced death anxiety in patients with gynecological cancer (Hajıhasanı and Naderı 2021). In another study, it was argued that religious attitudes had positive impacts on psychological adjustment in gynecological cancer treatment and that spiritual needs must be included in traditional cancer care processes (Duman et al. 2018). In a study examining the spiritual needs of family members who cared for cancer patients, it was reported that the patients performed prayers, supplications, and Qur’an reading activities, and that the spiritual need they felt most was friendship (Kıyancıçek and Çaydam 2017). It has also been reported to reduce anxiety and depression, slow down the development of cancer, and improve quality of life (Breitbart et al. 2010; Frost et al. 2012).

It was reported in the literature that cancer patients have a good level of social support (Ciria-Suarez et al. 2021; Düzen and Göktaş 2021; Social et al. 2022; Yesiana 2022). Effective social support systems have positive impacts both on the individual who gives support and on the person who receives that support. Family, friends, healthcare staff, social service organizations, and nongovernmental organizations are among the social support sources that individuals who are diagnosed with cancer can benefit from. In this respect, the quality, effectiveness, and strength of the support felt by these support systems are of great importance. Inadequate social support indicates a lack of psychological support, which can prevent psychological adaptation to cancer (Kaykunoğlu and Tambağ 2022).

Cancer is not only a physical illness but also a multidimensional experience affecting psychological, social, and existential domains. From an existential and stress–coping perspective, confrontation with a life-threatening diagnosis may intensify death anxiety, prompting individuals to mobilize both internal and external coping resources. SWB, as an internal resource, may provide meaning, hope, and existential coherence, thereby reducing death-related fears. Perceived social support, as an external resource, functions as a protective factor that buffers stress and enhances adaptive coping. Moreover, social support may reinforce SWB by fostering connectedness and emotional reassurance. Therefore, SWB and perceived social support may not only independently influence death anxiety but also interact in shaping psychological adjustment. However, the interrelationships among these variables remain insufficiently clarified, particularly among women with gynecologic cancer.

Nurses can identify spiritual needs, provide social support, and teach evidence-based coping strategies to reduce death anxiety by providing holistic care in the process of providing care to cancer patients. Despite increasing recognition of psychosocial and spiritual dimensions in oncology care, empirical evidence regarding the interplay between death anxiety, SWB, and perceived social support remains limited, especially in gynecologic oncology populations. The present study will help fill this gap in the literature and guide healthcare staff for comprehensive education and clinical practices for cancer patients. The present study aimed to uncover the relationship between death anxiety, SWB, and social support in gynecological cancer patients.

### Study questions

(1) What is the level of death anxiety in patients who are diagnosed with gynecological cancer? (2) What is the level of SWB in patients who are diagnosed with gynecological cancer? (3) What are the perceived social support levels of patients who are diagnosed with gynecological cancer? (4) Is there a relationship between death anxiety, SWB, and perceived social support levels of patients who are diagnosed with gynecological cancer?

## Methods

### Sample and design

The present study, which had a descriptive cross-sectional design, was conducted to determine the relationship between death anxiety, SWB, and social support of patients who had gynecological cancer. The study was conducted in the Gynecological Oncology Clinic of a university hospital between December 2023 and September 2024.

### Inclusion criteria

- Being 18 years of age or older
- Having been diagnosed with gynecological cancer at least 6 months ago
- Knowing and speaking Turkish

### Exclusion criteria

- Documented cognitive impairment that could interfere with questionnaire completion
- Diagnosed with a psychiatric disorder affecting comprehension
- Severe medical or neurological conditions preventing participation

The sample size was determined based on the results reported by Feng et al. (2021) using the Death Anxiety Scale scores. A power analysis was conducted using G*Power program. With an effect size of 0.11, power of 80%, and alpha level of 0.05, the required sample size was calculated as 513 participants.

Participants were recruited using a convenience sampling method. As this was a single-group descriptive study, the minimum required sample size was accepted as 513 participants. Considering potential data loss, 524 women with gynecologic cancer were approached. Five patients declined participation, and the study was completed with 519 participants.

### Measurements

The data were collected by the researcher through face-to-face interviews in the units of the specified hospital. Each interview lasted an average of 20 minutes.

The Personal Information Form, Death Anxiety Scale, Spiritual Well-being Scale, and Multidimensional Perceived Social Support Scale were used to collect the study data.

#### Personal Information Form

The Personal Information Form was developed by the researchers based on a review of studies examining sociodemographic and clinical characteristics associated with psychosocial outcomes in women with gynecologic cancer (Mansori et al. 2018; Chen et al. 2021; Feng et al. 2021; Var and Nazik 2024). The form consisted of 13 items, including age, marital status, education level, employment status, income level, having children, family type, diagnosis, time since diagnosis, and treatment modality.

#### Death Anxiety Scale

The scale was developed by Templer (1970) to be used to measure the level of death anxiety. The first adaptation of the Death Anxiety Scale to Turkish was performed by Şenol (1989), and the Cronbach alpha value for internal consistency was calculated as 0.72. The study investigating the validity and reliability of the Turkish translation of the scale by reviewing it in different groups in Turkish norms was conducted by Akça and Köse (2008), and the Cronbach alpha value was reported to be 0.75. The scale, which has a 2-point Likert style (True-False), consists of a total of 15 items, each of which is scored as “0” or “1.” The sum of the scores obtained from the scale gives the death anxiety score. The highest score that can be obtained from the scale is 15. As the score obtained from the scale increases, the level of death anxiety also increases. If the total score is 7 and above, it indicates that the person has high death anxiety (Akça and Köse 2008). In this study, the Cronbach alpha value of the scale was found to be 0.61.

#### Spiritual Well-Being Scale

The scale was developed by Peterman et al. (2002) to determine the SWB of individuals with cancer and other chronic diseases. The validity and reliability study in our country was conducted by Aktürk et al. (2017). The scale had a 5-point Likert style and consisted of 12 items scored between “0” and “4” (0-Not at all, 4-A lot). The scale consists of 3 subdimensions. The meaning subdimension (Items 2, 3, 5, 8) has a total score of 0–16; the peace subdimension (Items 1, 4, 6, 7) has a total score of 0–16; the belief subdimension (Items 9, 10, 11, 12) has a total score of 0–16; and the total scale score is 48 points. The sum of the scores obtained from the subdimensions determines the score that can be obtained from the scale. A higher scale score indicates better SWB. In the validity and reliability study of the scale, Cronbach’s alpha value was found to be 0.80 (Aktürk et al. 2017). In this study, Cronbach’s alpha of the scale was found to be 0.77 in the total score.

#### Multidimensional Scale of Perceived Social Support

The scale was developed by Zimet et al. (1988), and its Turkish validity and reliability were first established by Eker and Arkar (1995). The scale consists of 12 items and 3 subdimensions. It includes 3 groups, each consisting of 4 items, regarding the source of support (family, friend, and a special person). It has a 7-point Likert style. The lowest score that can be obtained from the entire scale is 12, and the highest score is 84. A high score indicates that perceived social support is high. In the validity and reliability study of the scale, Cronbach’s alpha value was found to be 0.89 (Eker et al. 2001). In this study, Cronbach’s alpha value of the scale was found to be 0.94 in the total score.

The validity and reliability of the measurement tools were supported by previously published Turkish adaptation studies. In addition, internal consistency reliability was reassessed in the present sample using Cronbach’s alpha coefficients.

### Statistical analysis

The data were analyzed using the SPSS software version 26.0. Kurtosis and skewness tests were used to evaluate the data for normal distribution. Percentage, arithmetic mean, and standard deviation tests were used to examine the sociodemographic and disease-related characteristics of the patients. The Pearson correlation analysis was used to determine the relationship between death anxiety, SWB, and multidimensional perceived social support. Before multiple regression analysis, the relationships between independent variables (descriptive characteristics) and dependent variables (death anxiety, SWB, and multidimensional perceived social support) were examined. Independent variables that were significantly associated with death anxiety, SWB, and multidimensional perceived social support were accepted as candidate predictors and entered by using the stepwise method. The separate multivariate regression analysis was made by using death anxiety, SWB, and multidimensional perceived social support as dependent variables. The linearity between the factors was analyzed to remove related variables from the model. In the preliminary model, all potential factors were included in the model and *R*^2^ was calculated. Estimates of model parameters and standard errors of these estimates were calculated. Statistical significance was taken as *p* < 0.05.

## Results

The sociodemographic characteristics of the participants are presented in Table 1. The majority of patients (63%) were aged between 45 and 65 years, 62% were married, 40.8% were primary school graduates, and 68.6% reported income lower than expenses. Additionally, 74.4% had children, and 65.9% lived in a nuclear family.

**Table 1.** The distribution of the sociodemographic characteristics of the patients (n = 519) Table 1 long description.

Variable	*n* (%)
Age	
Under 45 years	91 (17.5)
45–65 years	327 (63.0)
≥66 years	101 (19.5)
Mean age (years)	55.35 ± SD
Place of living	
City	255 (49.1)
District	195 (37.6)
Village	69 (13.3)
Marital status	
Single	197 (38.0)
Married	322 (62.0)
Educational status	
Illiterate	56 (10.8)
Literate	73 (14.1)
Primary school	212 (40.8)
Middle/high school	156 (30.1)
Bachelor’s degree or above	22 (4.2)
Working status	
Working	99 (19.1)
Unemployed	420 (80.9)
Income level	
Income less than expenses	356 (68.6)
Income equals expenses	163 (31.4)
Having children	
Yes	386 (74.4)
No	133 (25.6)
Family type	
Living alone	80 (15.4)
Nuclear family	342 (65.9)
Extended family	97 (18.7)

Disease-related characteristics are summarized in Table 2. Ovarian cancer was the most common diagnosis (36.6%), followed by corpus uteri cancer (32.6%) and cervical cancer (25.4%). Nearly half of the patients (49.5%) had been diagnosed 13–24 months prior to the study. The most frequent treatment modality was surgery combined with chemotherapy (57.2%). Additionally, 52.6% of the participants reported having at least 1 chronic disease.

**Table 2.** The distribution of the disease-related characteristics of the patients (n = 519) Table 2 long description.

Variable	*n* (%)
Type of disease	
Corpus uteri cancer	169 (32.6)
Cervical cancer	132 (25.4)
Ovarian cancer	190 (36.6)
Vulvar cancer	28 (5.4)
Time since diagnosis	
6–12 months	158 (30.5)
13–24 months	257 (49.5)
≥25 months	104 (20.0)
Treatment modality	
Surgery	129 (24.9)
Surgery + chemotherapy	297 (57.2)
Surgery + radiotherapy	93 (17.9)
Presence of chronic disease	
Yes	273 (52.6)
No	246 (47.4)
Type of chronic disease (*n* = 273)	
Hypertension (HT)	104 (38.1)
Diabetes mellitus (DM)	122 (44.7)
Asthma/COPD	54 (19.8)
Other	42 (15.4)

Descriptive statistics for the study variables are presented in Table 3. The mean Death Anxiety Scale score was 6.8 ± 2.95, indicating moderate death anxiety. The mean Spiritual Well-Being Scale score was 31.16 ± 5.24, and the mean Multidimensional Perceived Social Support Scale score was 62.46 ± 13.7.

**Table 3.** Descriptive statistics of scale and subdimension scores (n = 519) Table 3 long description.

Scale/subdimension	Possible score range	Observed score range	Mean ± SD
Death Anxiety Scale	0–15	0–14	6.8 ± 2.95
Spiritual Well-Being Scale	0–48	14–43	31.16 ± 5.24
Meaning	0–16	0–15	9.03 ± 2.17
Peace	0–16	0–15	9.07 ± 2.21
Belief	0–16	6–16	13.05 ± 2.33
Multidimensional Perceived Social Support Scale	12–84	12–84	62.46 ± 13.7
Family	4–28	4–28	20.11 ± 4.93
Friend	4–28	4–28	21.59 ± 5.16
Special person	4–28	4–28	20.75 ± 4.86

Pearson correlation analysis revealed a statistically significant positive correlation between Spiritual Well-Being Scale scores and Multidimensional Perceived Social Support Scale scores (*p* < 0.001). Positive correlations were also observed between all SWB subdimensions and social support subdimensions (*p* < 0.001). No statistically significant correlation was found between Death Anxiety Scale scores and SWB or perceived social support scores (*p* > 0.05) (Table 4).

**Table 4.** Correlations between scale and subdimension scores (*n* = 519) Table 4 long description.

Variables	1	2	3	4	5	6	7	8	9
1. SWB – meaning	1								
2. SWB – peace	0.622***	1							
3. SWB – belief	0.349***	0.279***	1						
4. SWB – total	0.832***	0.804***	0.709***	1					
5. MSPSS – family	0.272***	0.219***	0.385***	0.377***	1				
6. MSPSS – friend	0.367***	0.315***	0.505***	0.511***	0.612***	1			
7. MSPSS – special person	0.353***	0.316***	0.475***	0.491***	0.829***	0.837***	1		
8. MSPSS – total	0.362***	0.310***	0.498***	0.503***	0.885***	0.894***	0.969***	1	
9. Death anxiety – total	0.008	0.042	−0.041	0.003	−0.055	−0.014	−0.077	−0.052	1

*** *p* < .001.

Multiple regression analysis was performed to identify variables associated with SWB and perceived social support. Variables that showed significant associations in bivariate analyses were included as candidate predictors. In the regression model predicting SWB, the friend and special person subdimensions of the Multidimensional Perceived Social Support Scale were identified as significant predictors (*p* < 0.05). The model explained 27.4% of the variance in SWB scores (*R*^2^ = 0.274).

In the regression model predicting perceived social support, SWB total score and Belief subdimension score were found to be significant predictors (*p* < 0.05).

The model explained 29.3% of the variance in perceived social support scores (*R*^2^ = 0.293).

No statistically significant regression model was identified for death anxiety scores. Neither SWB nor perceived social support variables significantly predicted death anxiety (*p* > 0.05).

Regression analysis indicated that Friend and Special Person social support subdimensions were significantly associated with SWB scores. Additionally, SWB total and belief subdimension scores were significantly associated with perceived social support (Table 5).

**Table 5.** Multiple regression analyses predicting spiritual well-being and perceived social support (*n* = 519) Table 5 long description.

Dependent variable	Predictor	*B*	SE	*β*	*t*	*p*
Spiritual well-being	Constant	19.116	0.889	–	21.514	<0.001
	MSPSS – friend	0.338	0.070	0.332	4.847	<0.001
	MSPSS – special person	0.229	0.074	0.213	3.103	0.002
Perceived social support	Constant	16.190	3.213	–	5.039	<0.001
	SWB – total	0.787	0.137	0.301	5.737	<0.001
	SWB – belief	1.666	0.308	0.284	5.417	<0.001

## Discussion

This study examined death anxiety, SWB, and perceived social support in women with gynecologic cancer.

The findings indicated that participants experienced moderate levels of death anxiety, while SWB and perceived social support levels were relatively high. Additionally, although no statistically significant relationships were identified between death anxiety and the other variables, significant associations emerged between SWB, and perceived social support.

The mean Death Anxiety Scale score suggested that women with gynecologic cancer experienced a moderate degree of death anxiety. This finding is consistent with previous studies conducted in gynecologic oncology populations (Mansori et al. 2018; Feng et al. 2021), where death anxiety was similarly reported at moderate levels. Unlike our study, studies conducted on breast cancer patients reported higher levels of death anxiety (Rong et al. 2023; Ahmead et al. 2024). In contrast, studies involving breast cancer patients have documented higher death anxiety levels (Rong et al. 2023; Ahmead et al. 2024). These discrepancies may be explained by differences in disease perception, body image concerns, treatment experiences, and sociocultural meanings attributed to specific cancer types. Moderate death anxiety observed in this study may reflect patients’ awareness of the life-threatening nature of cancer while still maintaining a degree of psychological adaptation. This interpretation aligns with literature suggesting that death anxiety does not always manifest at extreme levels and may fluctuate depending on coping mechanisms, support systems, and illness trajectory (Wanglin 2024).

Participants demonstrated relatively high SWB scores, indicating that spirituality may serve as an important psychosocial resource in coping with gynecologic cancer. This finding is in agreement with prior oncology studies reporting moderate to high levels of SWB (Ata and Kılıç 2021; Chen et al. 2021; Kavalalı Erdoğan and Koç 2021). Belief subdimension scores were higher than other subdimensions, suggesting that faith-related coping mechanisms may play a particularly central role. Similar findings have been reported in Turkish oncology populations, where religious belief and spirituality were closely linked with psychological adjustment and resilience (Kurt 2021; Özdoğan 2021). SWB has been associated with improved emotional regulation, meaning-making, and adaptation to life-threatening illnesses (Chaar et al. 2018; Chen et al. 2021). In this context, higher SWB scores may indicate that participants utilized existential and faith-based frameworks to interpret and manage their illness experiences.

The high perceived social support scores observed in this study are consistent with earlier research conducted among oncology patients (Düzen and Göktaş 2021; Kaykunoğlu and Tambağ 2022; Budak and Ay Kaatsız 2024). Social support is widely recognized as a protective factor that buffers psychological distress, enhances coping capacity, and contributes to improved quality of life.

Strong family ties, cultural caregiving norms, and relational dynamics may explain elevated social support perceptions in this sample. Previous Turkish studies similarly highlight the importance of familial and interpersonal support in cancer adaptation (Balkan and Oskay 2023).

Contrary to expectations, no statistically significant relationships were identified between death anxiety and either SWB or perceived social support. While some previous studies have reported inverse relationships between death anxiety and SWB (Feng et al. 2021; Hajıhasanı and Naderı 2021), inconsistent findings have also been documented.

Several factors may account for this lack of association. Death anxiety is a complex construct influenced by multiple psychological, cultural, and situational variables. Differences in disease stage, prognosis perception, coping strategies, and existential orientation may alter how death anxiety interacts with spirituality and support systems (Wanglin 2024).

The study also found that there were no relationships between the level of social support, SWB, and death anxiety. Unlike the results of the present study, a qualitative study conducted in Turkey with women with gynecological cancer reported that high death anxiety could cause distress in patients and lead them to seek SWB. The same study also reported that spiritual resources such as religious practices and family support play an important role in alleviating anxiety and providing emotional stability (Rong et al. 2023). In the study by Yasmin et al., it was reported that increased death anxiety in women with gynecological cancer negatively affects SWB and that emotional-spiritual needs are as important as physical treatment (Mansori et al. 2018). The literature shows that increasing spiritual health improves general well-being and that managing death anxiety can increase the quality of life of oncology patients (Feng et al. 2021). Also, a meta-analysis has shown that death anxiety is positively associated with the female gender and negatively associated with SWB (Wanglin 2024). The reason why the study finding is different from the literature may be because most of the women participating in this study were within the first 2 years of diagnosis, and individual differences.

In the present study, the factors that best explained the total Spiritual Well-being Scale score were the Multidimensional Perceived Social Support Friend and Special Person subdimensions. Also, the factors that best explained the Multidimensional Perceived Social Support score were the total SWB and SWB belief subdimension. The findings show that SWB and social support are among the factors that best explain each other. This is similar to the studies in the literature (Tangwei et al. 2021; Li et al. 2022; Rong et al. 2023). Our findings show that SWB and social support are basic elements complementing each other and that an increase in one supports the other and contributes significantly to the general well-being of patients with gynecological cancer.

## Limitations

This study has several limitations that should be considered when interpreting the findings. Due to its cross-sectional design, causal relationships between death anxiety, SWB, and perceived social support cannot be established. Although significant associations were identified, the direction and temporality of these relationships remain unclear. Longitudinal or prospective studies are needed to better understand causal pathways and changes over time.

The research was conducted in a single tertiary hospital in Adana, Turkey, which may limit the generalizability of the findings to other geographic regions, healthcare systems, and cultural contexts. Cultural, religious, and social norms may substantially influence spirituality, perceptions of social support, and attitudes toward death, potentially restricting the transferability of the results to more diverse populations.

All variables were assessed using self-report instruments. Constructs such as spirituality and death anxiety are inherently sensitive and subjective, increasing the risk of response bias, recall bias, and social desirability effects. Participants may have underreported distress or overreported SWB and perceived social support.

Although the sample size was relatively large, the study population included patients with different gynecologic cancer types, treatment modalities, and likely varying disease stages. Clinical variables such as stage of disease, recurrence status, symptom burden, treatment intent (curative vs. palliative), and psychiatric comorbidities were not controlled for in the analyses. These factors may have influenced levels of death anxiety and psychosocial outcomes.

In addition, other psychological constructs known to be associated with death anxiety in oncology populations – such as depression, hopelessness, coping styles, resilience, or meaning in life – were not evaluated. The absence of these variables may have limited the explanatory power of the regression analyses.

Finally, the study was not conducted exclusively within a palliative or end-of-life care context. Therefore, the findings may not fully reflect the experiences of patients receiving specialized palliative care or those with advanced-stage disease.

## Conclusion and recommendations

In the present study, it was found that the level of death anxiety was at a moderate level in gynecological cancer patients, and the levels of SWB and perceived social support were high. The level of SWB increased as the level of perceived social support increased. It was also found that there was no relationship between the level of perceived social support and SWB and death anxiety. Based on these results, it can be suggested that a multidisciplinary approach should be adopted in holistic care to meet the spiritual needs of gynecological cancer patients. In addition, programs should be organized to increase the support of the family and social environment toward patients, reduce the emotional burden of the patients by strengthening the family support, and improve the well-being of the patients by providing psychosocial support programs and spiritual counseling services.

## Supporting information

10.1017/S1478951526102727.sm001 Sezer Daşçikaran and Nazik supplementary material Sezer Daşçikaran and Nazik supplementary material

## Data Availability

The data that support the findings of this study are available from the first author upon reasonable request.

## Supplementary material

The supplementary material for this article can be found at https://doi.org/10.1017/S1478951526102727.

## Table 1 Long description

The table presents socio-demographic characteristics of 519 patients, highlighting age, living place, marital status, education, employment, income, children, and family type. A majority, 63%, are aged between 45 and 65 years, with a mean age of 55.35 years. Nearly half reside in cities, and 62% are married. Educationally, 40.8% completed primary school, while only 4.2% have a bachelor's degree or higher. Most patients, 80.9%, are unemployed, and 68.6% report their income is less than their expenses. A significant portion, 74.4%, have children, and 65.9% live in nuclear families. These data suggest a trend of middle-aged, city-dwelling, married individuals with lower educational attainment and financial challenges.

Navigate back to Table 1.

## Table 2 Long description

The table details disease-related characteristics of 519 patients, focusing on cancer type, time since diagnosis, treatment modality, and presence of chronic diseases. Ovarian cancer is the most prevalent at 36.6%, followed by corpus uteri cancer at 32.6%. The majority of patients, 49.5%, were diagnosed 13 to 24 months ago. Surgery combined with chemotherapy is the most common treatment, used by 57.2% of patients. Over half of the patients, 52.6%, have a chronic disease, with diabetes mellitus being the most common at 44.7% among those with chronic conditions. The data highlights the diversity in disease types and treatment approaches, with a significant portion of patients managing chronic diseases alongside cancer.

Navigate back to Table 2.

## Table 3 Long description

This table presents descriptive statistics for various scales and subdimensions related to well-being and social support among 519 participants. The Spiritual Well-Being Scale has a high mean score of 31.16, suggesting a generally positive spiritual state, while the Death Anxiety Scale shows a lower mean of 6.8, indicating moderate anxiety levels. Subdimensions of spiritual well-being, such as Meaning, Peace, and Belief, have similar observed score ranges, with Belief showing the highest mean score of 13.05. The Multidimensional Perceived Social Support Scale has a wide possible score range, with a mean of 62.46, reflecting strong perceived social support. Family, Friend, and Special Person subdimensions have similar mean scores, indicating balanced support across these areas. Observed score ranges are narrower than possible ranges, suggesting some limitations in score variability.

Navigate back to Table 3.

## Table 4 Long description

The table presents correlations between subjective well-being (SWB) dimensions, multidimensional scale of perceived social support (MSPSS) dimensions, and death anxiety among 519 participants. The MSPSS Total score has the highest correlation with MSPSS Special Person at 0.969, indicating a strong relationship. SWB Total is strongly correlated with SWB Meaning and SWB Peace, with values of 0.832 and 0.804 respectively. In contrast, Death Anxiety Total shows weak correlations with all other variables, with the highest being 0.042 with SWB Peace. These findings suggest that perceived social support, especially from a special person, is closely linked to overall social support, while death anxiety is relatively independent of the other measured dimensions.

Navigate back to Table 4.

## Table 5 Long description

The table presents multiple regression analyses predicting spiritual well-being and perceived social support among 519 participants. For spiritual well-being, support from friends and a special person are significant predictors, with standardized coefficients of 0.332 and 0.213, respectively. For perceived social support, overall spiritual well-being and belief are significant predictors, with standardized coefficients of 0.301 and 0.284, respectively. All predictors are statistically significant with p-values less than 0.01. The constant values for spiritual well-being and perceived social support are 19.116 and 16.190, respectively, indicating baseline levels when predictors are zero. The analysis suggests that both social support and spiritual factors are important in predicting well-being outcomes.

Navigate back to Table 5.
