# Supplementary material for: Relationship between death anxiety, spiritual well-being, and social support in patients with gynecologic cancer
Source: Palliat Support Care. 2026 Jun 3;24:e153. doi: 10.1017/S1478951526102727 (PMC13244346; doi:10.1017/S1478951526102727)
Supplement: Sezer Daşçikaran and Nazik supplementary material [file S1478951526102727sup001.doc]

Dear Editor,

I am submitting to our article “**Relationship between Death Anxiety, Spiritual Well-being, and Social Support in Patients with Gynecologic Cancer”.** And, we have no financial or other interest in the product or distributor of the product and any relation between ourselves and the manufacturer or distributor of the product.

The manuscript, including related data, figures and tables has not been previously published and is not under consideration elsewhere and will not be published elsewhere while under consideration by ***Palliative & Supportive Care.*** We give our permission to the Publisher to use any published material.

The authors have no commercial associations or sources of support that might pose a conflict of interest. All authors have made substantial contributions to the conception and design of the study, acquisition of data, or analysis and interpretation; drafting the article or revising it critically for important intellectual content and final approval of the version to be submitted. All authors endorse the data and conclusions. If there is any improvements or changes that you would like us to make please let us know.

Finally, the whole text has been translated and edited by a professional translation center. The certificate presented by the professional translation center has been delivered to you.

We look forward to hearing from you soon.

Faithfully Yours
